# Supplementary material for: Energy storage in structural composites by introducing CNT fiber/polymer electrolyte interleaves
Source: Sci Rep. 2018 Feb 21;8:3407. doi: 10.1038/s41598-018-21829-5 (PMC5821880; doi:10.1038/s41598-018-21829-5)
Supplement: Supplementary file 1 — Supplementary information [file 41598_2018_21829_MOESM1_ESM.doc]

Supplementary Information

**Energy storage in structural composites by introducing CNT fiber/polymer electrolyte interleaves.**

Evgeny Senokos^a,b,c^, Yunfu Ou^a,b^, Juan Jose Torres^a^, Federico Sket^a^, Carlos González^a,b^, Rebeca Marcilla^c^, Juan Jose Vilatela^a^*

**
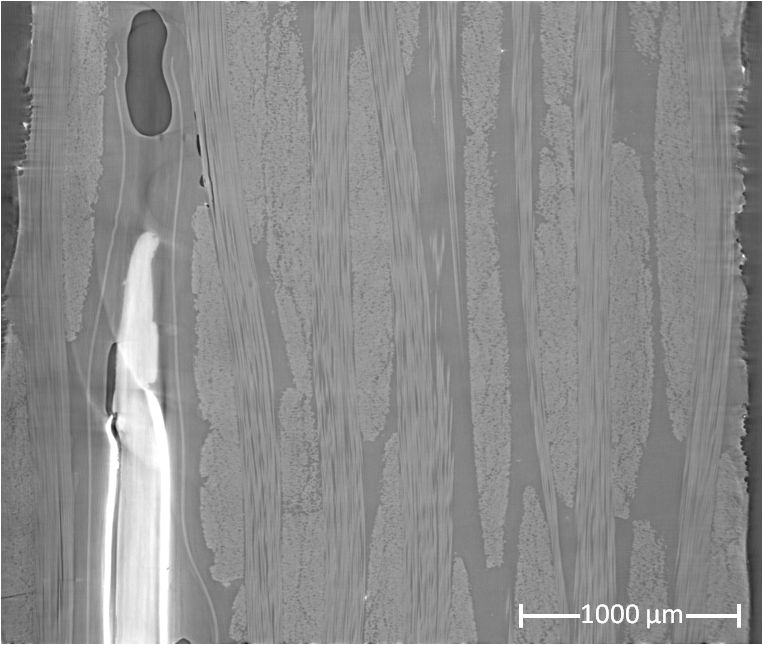
**

**Figure 1S.** Soft X-ray computed tomography image of cross-sectional area of structural SC showing voids at the interleaf edge. These voids are caused by the presence of the thin plastic tape used to electrically insulate the EDLC interleaf from the CF tows.

**
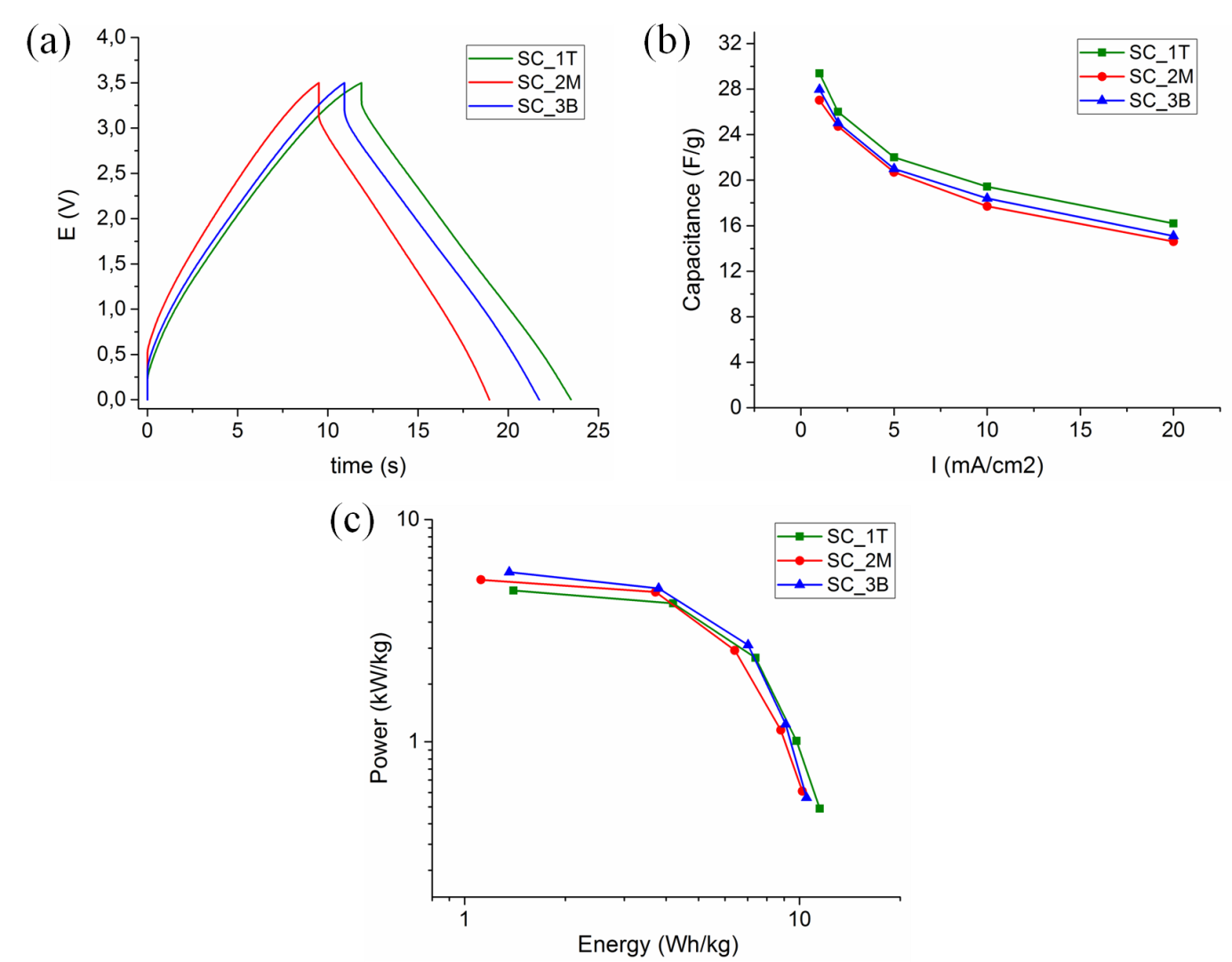

Figure 2S.** Electrochemical performance of the embedded EDLC interleaves. (a) CD profiles

measured at 5 mAcm^-2^, (b) specific capacitance and (c) Ragone plot obtained for SC_1T, SC_2M and SC_3B integrated into the structural composite.

**
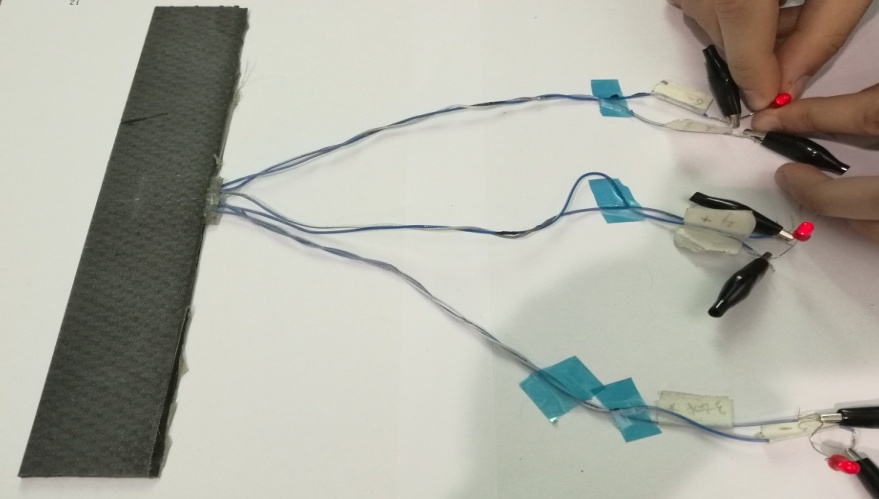
**

**Figure 3S.** Photograph of three EDLC device embedded into structural composite independently powering red LEDs.

**
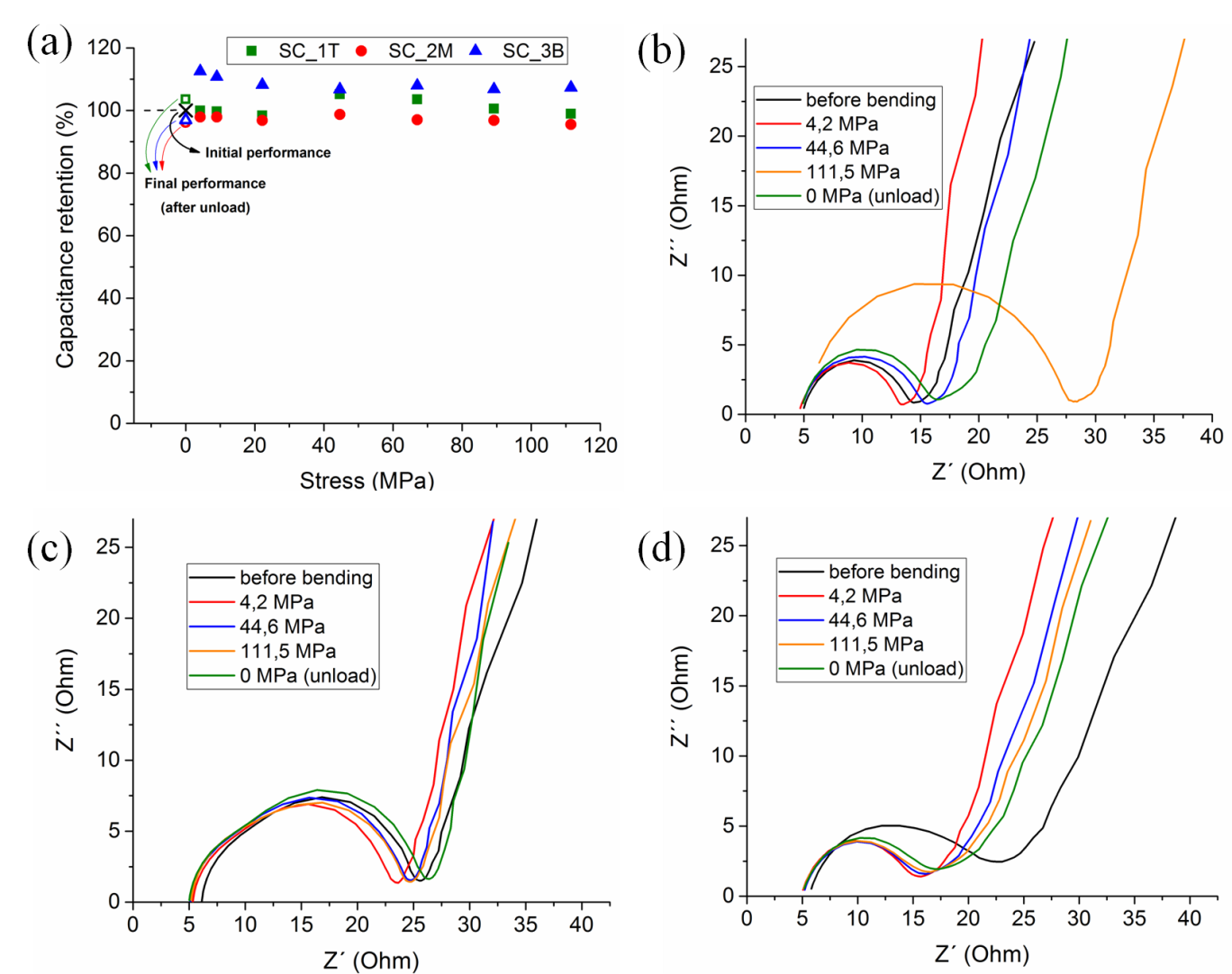

Figure 4S.** Electrochemical performance of the embedded EDLC devices. a) Specific capacitance obtained from CD profiles measured at 5 mAcm^-2^. Impedance plot for the different interleaves integrated into the structural composite during the bending test b) SC_1T, c) SC_2M and c) SC_3B.


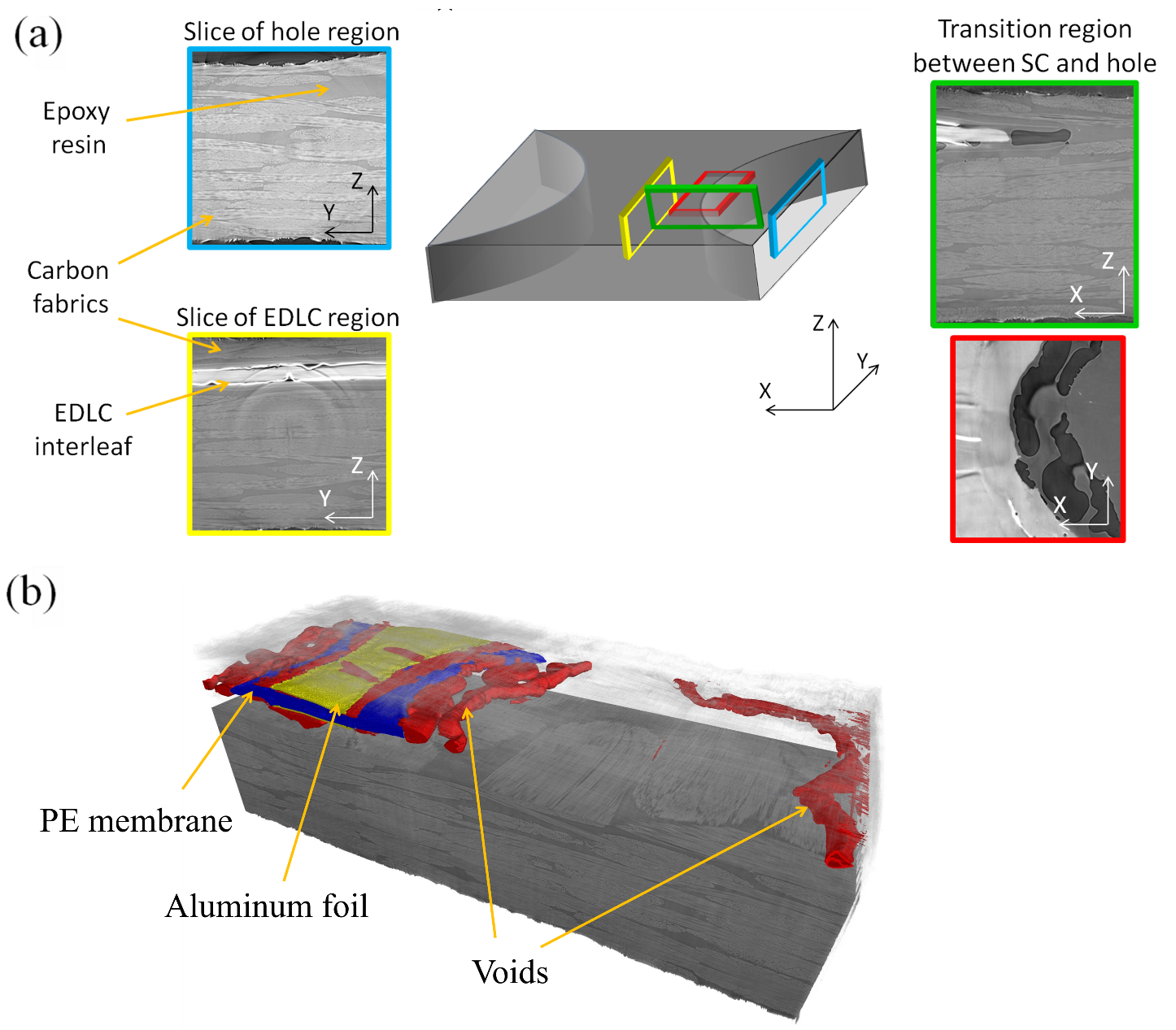


**Figure 5S.** High-resolution a) 2D and b) 3D tomography image of CFs/epoxy composite, confirming penetration of epoxy resin through channels in the interleaf and sowing the volume fraction of voids appearing at the edge of the EDLC.

**
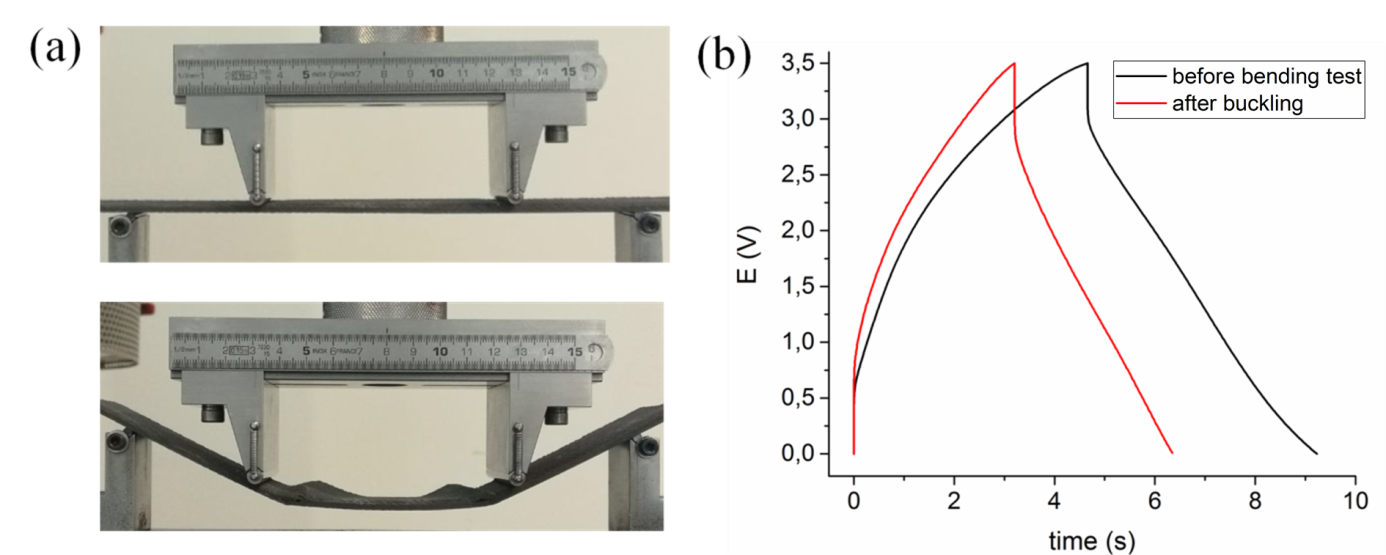

Figure 6S.** Electrochemical performance of structural SC before bending test and after buckling. (a) Photos of 4 point bending test setup and (b) Charge-discharge curves obtained before and after buckling of the structural SC.

**
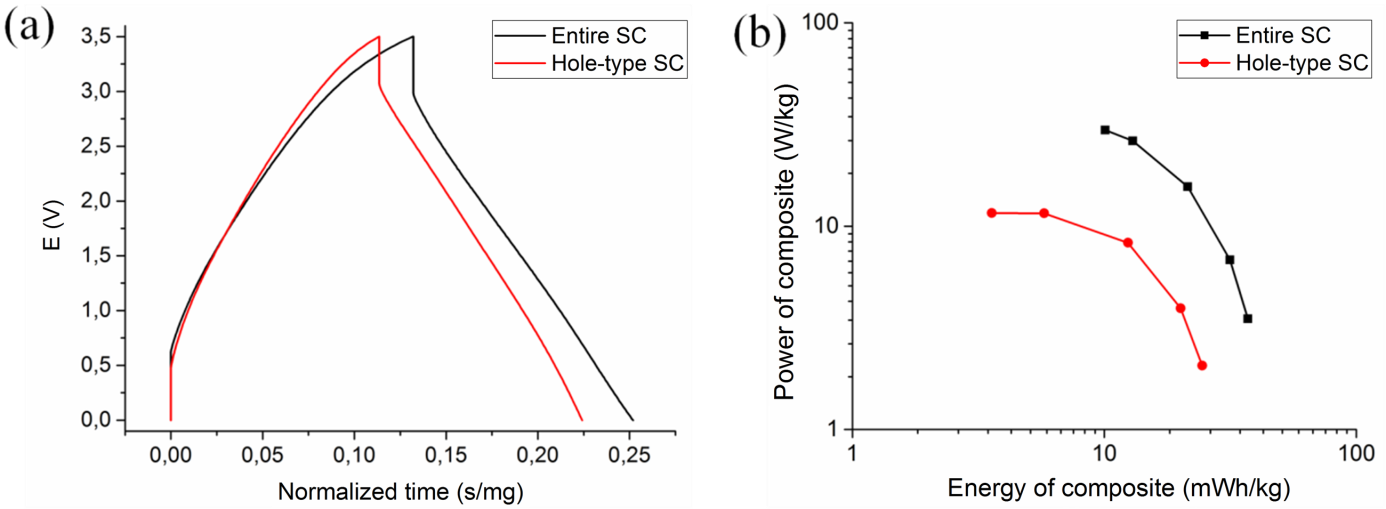

Figure 7S.** Comparison of electrochemical performance of regular 32 cm^2^ EDLC interleaf and a modified samples with grid structure containing 18 holes. (a) Charge-discharge profiles and (b) Ragone plots (energy and power are normalized by the total mass of composite) obtained for structural SCs containing entire and modified EDLC devices. The energy and power densities of the grid electrode are *pro rata* with effective area.

**Envelope of multifunctional properties.**

We explore analytically the envelope of multifunctional properties of the grid-shaped EDLC interleaf architectures.

Composite energy (E) and power (P) densities are given by


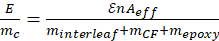
,

and


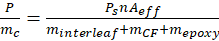
,

where *m_i_* is the mass of the different components, *n* the number of interleaves, *A_eff_* their effective planar area and ɛ and *P_s_* are energy and power densities per unit planar area, respectively. The planar area is simply the projected area of the device from top view. Trivially,
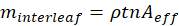
, where *ρ* is the ELDC volumetric density and *t* its thickness. Interlaminar properties such as shear modulus *G_12_* can be similarly expressed as a function of *A_eff_* as


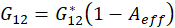


With equations 1-3 one can easily predict the energy density, power density and shear modulus of a range of interleaf designs, with properties following the behavior in Figure 8S.

**Figure 8S.** Predicted shear modulus (*G_12_*), a) Energy density and b) Power density as a function of the interleaf area. The prediction is for n = 1, t = 0.56 mm. Maximum shear modulus of 4.3 GPa corresponds to pure epoxy.

**Multifunctional performance analysis**

The ultimate weight-saving determined by multifunctional performance of the structural composite can be achieved through the balance between conflicting electrochemical and mechanical properties. Figure 9Sa shows the plot of energy density against longitudinal Young’s modulus calculated for different configurations of embedded EDLCs. Such representation enables to identify the key parameters to make multifunctional structures that produce weight reduction relative to monofunctional systems.

The black dashed line shows the combination of monofunctional systems considering a conventional Maxwell BCAP0010 (10 F) supercapacitor (2.9 Wh kg^-1^)^[1]^ as electrochemical device, and our control [0^o^]_8_ fiber reinforced polymer (E = 60 GPa) composite as a benchmark monofunctional structural component. Evidently, both energy and modulus are proportional to the mass fraction of its corresponding monofunctional component. The green line corresponds to the results of this work. While they are still below the level for weight reductions, it is of interest to analyse the effect of different plausible improvements to these materials on their multifunctional performance. Figure 9Sa shows, for example, that removal of aluminium current collectors (Al cc) and a reduction of polymer electrolyte (PE) membrane thickness (red curve) would produce very substantial improvements in performance, particularly for multifunctional composites with high energy density requirements. In contrast, an increase in the Young’s modulus of the CNT fiber fabrics to 30 GPa (blue curve), would not lead to large performance improvements. According to the analysis, a more promising method to improve multifunctional performance is to introduce pseucopacitive metal oxides in the CNT fiber fabric, which has been recently demonstrated to produce monofunctional materials with high energy density (e.g. MnO_2_).^[2]^ Coupled with optimization of membrane thickness, this would lead to a multifunctional composite material offering a weight reduction.

**
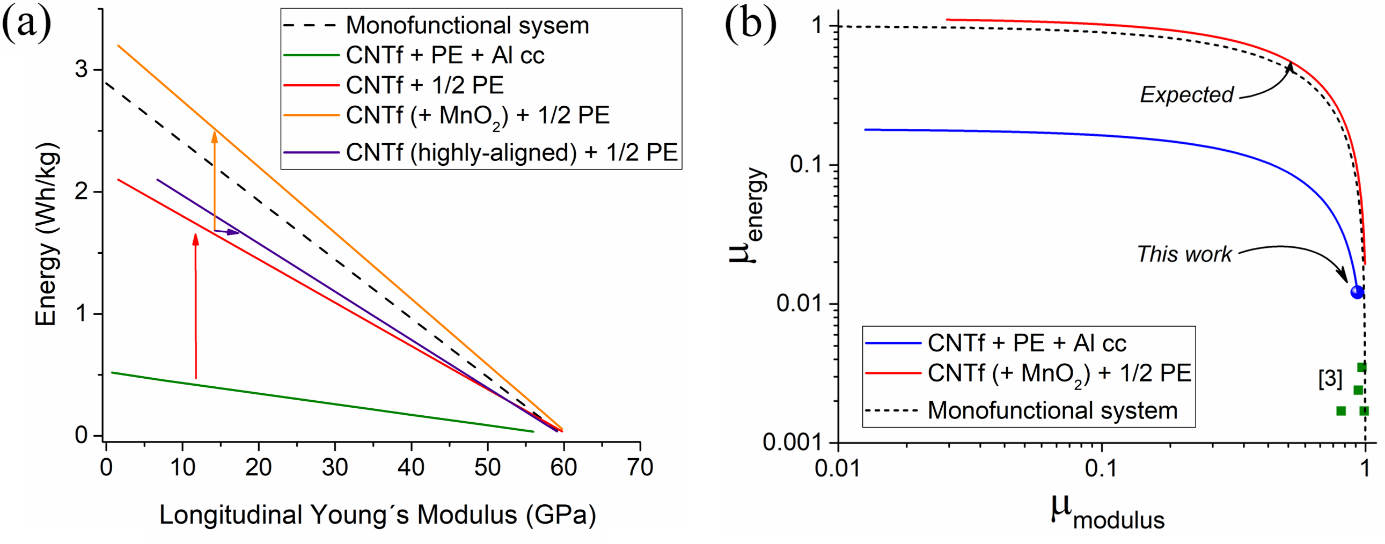

Figure 9S.** Multifunctional properties of structural power composites. (a) The plot of energy density vs Young’s modulus at different mass fraction of electrochemical component estimated for alternative EDLC’s configurations. (b) Multifunctional plot showing contribution of electrochemical and mechanical efficiencies in structural composites.

A more general approach to examine multifunctional performance of the structural power composites consists in calculating an efficiency index (µ_mf_) as the sum of the electrochemical and (µ_energy_ = Energy_(mf)_/Energy_(0)_) and mechanical (µ_modulus_ = E_(mf)_/E_(0)_) efficiencies with respect to the monofunctional materials. Here Energy_(mf)_ and Energy_(0)_ refer to energy density of multifunctional material and the conventional device, respectively, and E_(mf)_ and E_(0)_ to the longitudinal Young’s moduli of multifunctional and monofunctional composites, respectively. Weight reductions at a materials level correspond to µ_mf_ > 1.

The plot in Figure 9Sb compares the reference monofunctional system (black line) with different multifunctional materials. The use of EDLC interleaves based on CNT fibers and the PE noticeably raises the value of µ_energy_ while preserving µ_modulus_ close to 1, although with µ_mf_ currently at 0.95. But the analysis shows that integrating pseudocapacitive metal oxides and optimizing the interleaf device architecture will lead to µ_mf_ around 1.02 - 1.13. Very importantly, a key feature of the multifunctional architectures introduced in this work is that they enable tailoring composite properties by controlling the mass fraction of electrochemical and FRP components to meet a particular balance of mechanical and electrochemical contributions.

For reference, we include literature data corresponding to structural supercapacitor composites with CNT-grafted CF active material, GF fabrics separator and a multifunctional matrices based on IL and Li salt. These systems have mechanical efficiency near unity, but a relatively low energy density, leading to µ_mf_ around 0.82 - 1.00.^[3]^ However, these materials have very high compressive and shear moduli, and good prospects for increasing their energy density.^[4]^

**
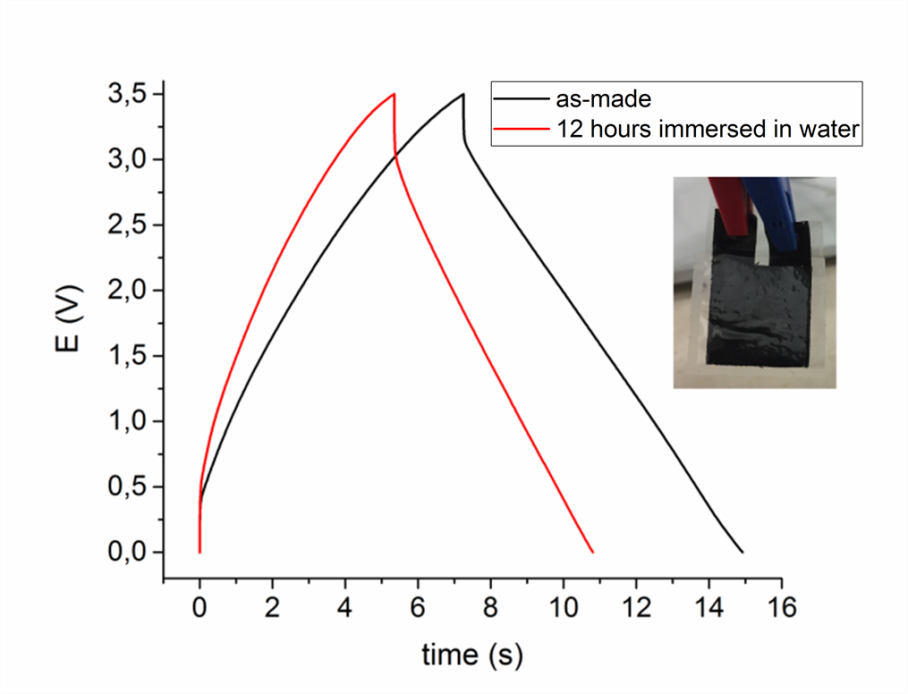
**

**Figure 10S.** Charge-discharge curves showing the negative effect of water on the electrochemical performance of free-standing EDLC device without encapsulation.

REFERENCES

(1) Maxwell HC Series ultracapacitors datasheet <http://www.maxwell.com/images/documents/hcseries_ds_1013793-9.pdf> [accessed 12.07.17].

(2) Pendashteh, A.; Senokos, E.; Palma, J.; Anderson, M.; Vilatela, J. J.; Marcilla, R. Manganese Dioxide Decoration on Macroscopic CNT Fibers: From High-Performance Liquid-Based to All-Solid-State Supercapacitors. *J. Power Sources* **2017**, *372*, 64-73.

(3) Greenhalgh, E. S.; Ankersen, J.; Asp, L. E.; Bismarck, A.; Fontana, Q. P. V; Houlle, M.; Kalinka, G.; Kucernak, A.; Mistry, M.; Nguyen, S. Mechanical, Electrical and Microstructural Characterisation of Multifunctional Structural Power Composites. *J. Compos. Mater.* **2015**, *49* (15), 1823–1834.

(4) Asp, L. E.; Greenhalgh, E. S. Structural Power Composites. *Compos. Sci. Technol.* **2014**, *101*, 41–61.
